# O068: Implementation of antimicrobial copper in neonatal intensive care unit (NICU)

**DOI:** 10.1186/2047-2994-2-S1-O68

**Published:** 2013-06-20

**Authors:** P Efstathiou, M Anagnostakou, E Kouskouni, C Petropoulou, K Karageorgou, Z Manolidou, S Papanikolaou, M Tseroni, E Logothetis, V Karyoti

**Affiliations:** 1 National Health Operations Centre, Athens, Greece; 2 “Agia Sophia” Children’s Hospital (NICU), Athens, Greece; 3 Medical School of the University of Athens, Microbiology laboratory of Aretaieio Hospital, Ministry of Health , Athens, Greece

## Objectives

The aim of this study was to investigate the effectiveness of the application of antimicrobial copper alloys (Cu +) in a Neonatal Intensive Care Unit (NICU) in relation to the reduction of microbial flora.

## Methods

At a Level III Neonatal Intensive Care Unit of a pediatric hospital, with the capacity of twenty-six (26) incubators, antimicrobial copper (Cu +) was implemented on touch surfaces and objects. The copper alloy contains Cu 63% - Zn 37% (Lead Low). Microbiological cultures were taken in three different time periods, before and after the application of Cu^+^, using dry and wet method technique.

## Results

In the above NICU, the reduction of microbial flora after the implementation of the antimicrobial copper (Cu +) on the selected surfaces and objects was statistically significant (n = 15, p <0,05) and was recorded at 90%. The pathogens isolated at high rates (CFU / ml) prior to copper implementation were as follows: Klebsiella spp., Staph. Epidermidis, Staph. Aureus, Enterococcus spp.

## Conclusion

This study highlights the positive impact of antimicrobial copper (Cu +) and demonstrates that copper implemented surfaces and objects are effective in neutralizing bacteria, which are responsible for Health Care Acquired Infections in the nosocomial environment (HCAIs).

The innovative implementation of antimicrobial copper in the NICU and the significant reduction of microbial flora heralds the reduction of antimicrobial drugs use, and a possible reduction of hospital acquired infections and hospitalization time.

## Disclosure of interest

None declared.

